# Tri-leaflet mitral valve in hypertrophic obstructive cardiomyopathy

**DOI:** 10.1007/s12055-024-01871-x

**Published:** 2024-12-17

**Authors:** André Alexandre, André Dias-Frias, Bruno Brochado, Mário Jorge Amorim, Patrícia Rodrigues, João Silveira, Isabel Sá, Sofia Cabral, Severo Torres

**Affiliations:** 1 Department of Cardiology, Unidade Local de Saúde de Santo António, Largo Do Prof. Abel Salazar, 4099-001 Porto, Portugal; 2 ICBAS – School of Medicine and Biomedical Sciences, University of Porto, Porto, Portugal; 3 Department of Cardiothoracic Surgery, Unidade Local de Saúde de São João, Porto, Portugal

**Keywords:** Hypertrophic obstructive cardiomyopathy, Tri-leaflet mitral valve, Multimodality imaging, Surgical myectomy

## Abstract

A tri-leaflet mitral valve (MV) is an extremely rare phenomenon, often associated with significant mitral regurgitation (MR). It may represent a phenotypic expression of hypertrophic obstructive cardiomyopathy (HOCM). We present the case of a 69-year-old woman with HOCM, a tri-leaflet MV, and systolic anterior motion (SAM)–induced severe MR. Despite receiving optimal medical therapy, her refractory symptoms and significant MR prompted surgical consideration. The patient underwent surgical myectomy, resulting in improvement of left ventricular outflow tract obstruction, SAM-induced MR, and symptomatic relief. This case illustrates a unique presentation of HOCM with a tri-leaflet MV and significant MR, successfully managed with a tailored surgical myectomy.

**Supplementary Information:**

The online version contains supplementary material available at 10.1007/s12055-024-01871-x.

## Background

A tri-leaflet mitral valve (MV) is an extremely rare phenomenon, with only nine reported cases to date [1–3]. The majority of these cases are associated with hypertrophic obstructive cardiomyopathy (HOCM) [2, 3]. In HOCM patients with a tri-leaflet MV, mitral regurgitation (MR) is often caused by systolic anterior motion (SAM) of the MV, leading to eccentric MR jets. We report the case of a patient with HOCM, a tri-leaflet MV, and severe MR who underwent successful septal surgical myectomy, which effectively reduced MR and patient’s symptoms.

## Case presentation

A 69-year-old woman was referred to the cardiology clinic due to dyspnea (New York Heart Association [NYHA] class III), angina with mild efforts, and exertional syncope. The patient’s medical history was relevant for left bundle branch block for over a decade and a prior history of grade 1 arterial hypertension, initially treated with medication, but discontinued in the past year due to the onset of syncopal episodes. Her family history was unremarkable for heart failure, sudden cardiac death, or coronary artery disease.

**Fig. 1:**
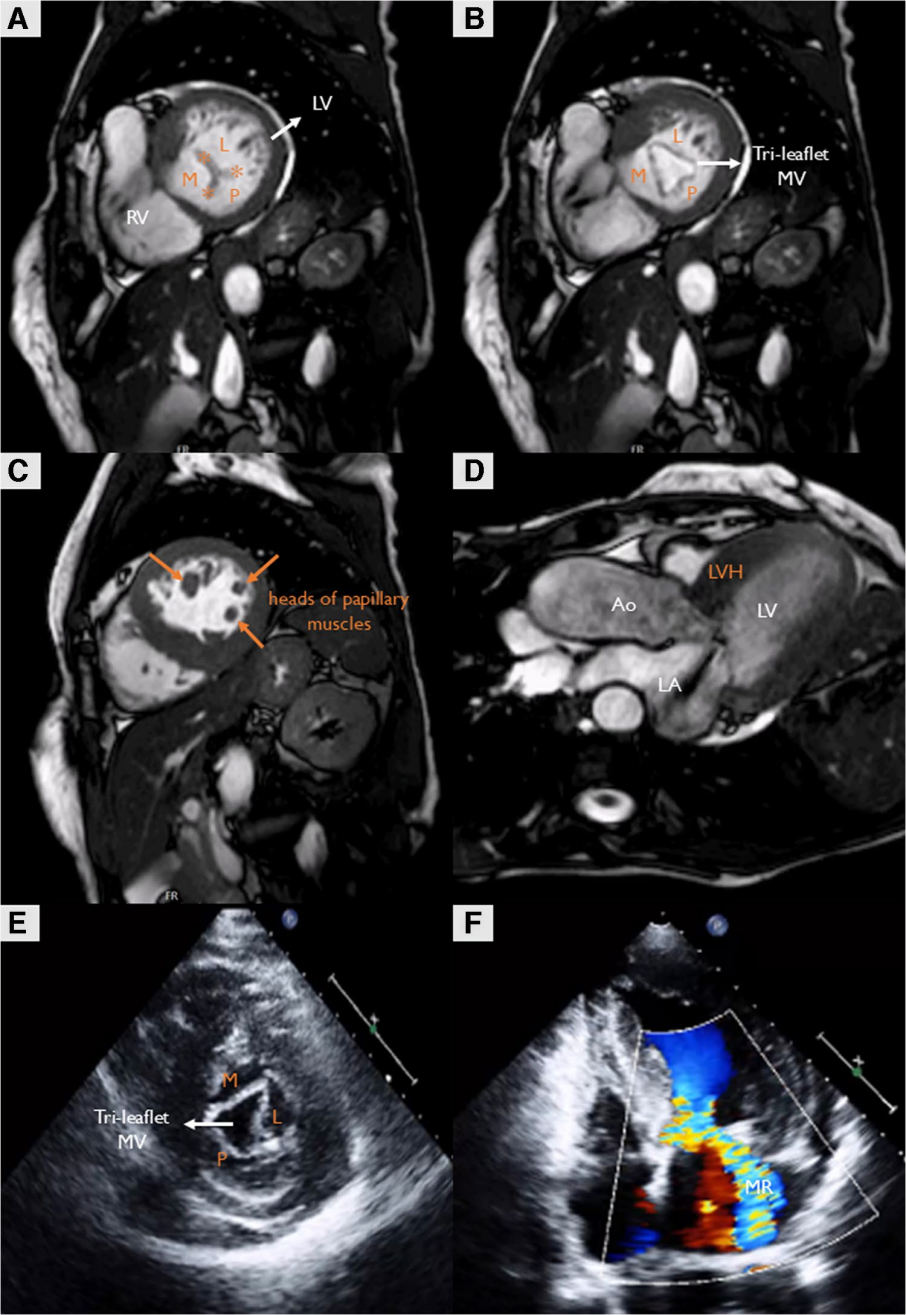
Multimodality imaging of a tri-leaflet MV in HOCM. **A**–**C** Still frames of cine CMR (steady-state free precession sequence; short-axis views), demonstrating a tri-leaflet MV in closed (**A**) and open (**B**) positions, with the respective three anteriorly displaced papillary muscles (**C**). **D** Still frame of cine CMR (steady-state free precession sequence; three-chamber view) showing systolic anterior motion of the MV, with an eccentric MR jet directed to the posterior wall of the LA. **E** TTE (parasternal short-axis view) depicting a tri-leaflet MV in the open position. **F** Baseline TTE (apical four-chamber view) showing a moderate to severe, eccentric MR jet directed towards the lateral wall of the LA. Ao, ascending aorta; CMR, cardiac magnetic resonance; HOCM, hypertrophic obstructive cardiomyopathy; L, lateral leaflet of the MV; LA, left atrium; LV, left ventricle; LVH, left ventricular hypertrophy; M, medial leaflet of the MV; MR, mitral regurgitation; MV, mitral valve; P, posterior leaflet of the MV; RV, right ventricle; TTE, transthoracic echocardiography. Asterisks (*) indicate the three equidistant commissures. Orange arrows indicate the heads of the three anteriorly displaced papillary muscles

Echocardiography revealed asymmetric septal left ventricular hypertrophy (18 mm), with SAM and left ventricular outflow tract obstruction (LVOTO) with peak intraventricular gradient (IVG) at rest of 85 mmHg, indicative of HOCM (Fig. 1 and S1, Video S1, S2). Notably, the MV exhibited an extremely rare tri-leaflet morphology, with anteriorly displaced papillary muscles, and SAM-related significant MR. Cardiac magnetic resonance (CMR) confirmed the HOCM phenotype and the presence of a noncleft tri-leaflet MV with three papillary muscles (Fig. 1, Video S3, S4). The diagnosis of a tri-leaflet MV was based on the identification of three equidistant commissures and a central coaptation point, along with three slightly anteriorly displaced papillary muscles, and concordant atrioventricular and ventriculoarterial connections, with no evidence of congenital abnormalities. In our patient, LVOTO and SAM-induced MR were the predominant mechanisms of symptoms. Despite receiving optimal medical therapy, the persistent refractory symptoms (NYHA III) and significant MR prompted surgical consideration. The patient underwent surgical myectomy, preserving the native MV. The 12-month follow-up demonstrated a successful result with symptomatic relief (NYHA II), resolution of LVOTO (peak IVG was 14 mmHg with Valsalva maneuver), and mild central MR (Fig. S2, Video S5).

The other family members were screened using echocardiography, but none was found to have hypertrophic cardiomyopathy. Genetic testing for the most common mutations associated with HOCM (MYH7, MYBPC3, TNNT2, TNNI3, TPM1, MYL2, MYL3) was negative in our patient. Additionally, screening for HOCM phenocopies, including urine and blood immunofixation, Fabry’s disease screening (which included polymerase chain reaction [PCR] and sequencing of the galactosidase alpha [GLA] gene, including the intronic variant c.639 + 919G > A), and technetium-99 m-3,3-diphosphono-1,2-propanodicarboxylic acid (99mTc-DPD) scintigraphy, was also negative.

## Conclusions

This case illustrates a unique presentation of HOCM with a tri-leaflet MV and severe MR, successfully managed with surgical myectomy. Our findings support the hypothesis that a tri-leaflet MV may represent a phenotypic expression of HOCM, as this condition encompasses not only increased thickness and hypercontractility but also abnormalities of the MV apparatus. Despite its rare and complex nature, the successful preservation of the tri-leaflet MV’s functionality through surgical myectomy underscores the importance of tailored interventions in managing this unique condition.

## Supplementary Information

Below is the link to the electronic supplementary material.

Supplementary file1 (TIF 2214 KB)

Supplementary file2 (TIF 2055 KB)

Supplementary file3 (MP4 4408 KB)

Supplementary file4 (MP4 6069 KB)

Supplementary file5 (MP4 17565 KB)

Supplementary file6 (MP4 2808 KB)

Supplementary file7 (MP4 6381 KB)

## Data Availability

The data that support the findings of this study are available from the corresponding author upon reasonable request.

## References

[1] Chui J, Anderson RH, Lang RM, Tsang W. The trileaflet mitral valve. Am J Cardiol. 2018;121:513–9. 10.1016/j.amjcard.2017.11.018. 29304994 10.1016/j.amjcard.2017.11.018

[2] Moya-Mur JL, Garcia-Martin A, Jimenez-Nacher JJ, Fernandez-Golfin C, Zamorano-Gomez JL. ‘Tri-leaflet mitral valve morphology’: a new phenotypic expression in hypertrophic cardiomyopathy? Eur Heart J Cardiovasc Imaging. 2015;16:692. 10.1093/ehjci/jev034. 25787745 10.1093/ehjci/jev034

[3] Castellani C, Smith AAH, Mohananey D, Garster N. Trileaflet mitral valve in the setting of hypertrophic cardiomyopathy: a curious rarity with a possible association. Circ Cardiovasc Imaging. 2022;15:e013772. 10.1161/CIRCIMAGING.121.013772. 35345893 10.1161/CIRCIMAGING.121.013772

